# 

**Published:** 1823-07

**Authors:** 


### LONDON MEDICAL AND PHYSICAL JOURNAL.

CONTAINING

ORIGINAL CORRESPONDENCE OF EMINENT PRACTITIONERS,

AND

CRITICAL ANALYSIS OF NEW WORKS

RELATING TO MEDICINE, SURGERY, MIDWIFERY, CHEMISTRY, PHARMACY, BOTANY, AND NATURAL HISTORY.

---

EDITED BY

RODERICK MACLEOD, M.D.

LICENTIATE OF THE COLLEGE OF PHYSICIANS OF LONDON; MEMBER OF THE MEDICO-CHIRURGICAL SOCIETY OF LONDON, AND OF THE ROYAL MEDICAL SOCIETY OF EDINBURGH; PHYSICIAN TO THE WESTMINSTER GENERAL DISPENSARY; TO THE INFIRMARY FOR CHILDREN, AND TO THE SCOTTISH HOSPITAL; LECTURER ON THE PRACTICE OF PHYSIC AND MATERIA MEDICA, AT THE MEDICAL THEATRE, WINDMILL-STREET:

AND

JOHN BACOT, Esq.

MEMBER OF THE ROYAL COLLEGE OF SURGEONS; AND LATELY SURGEON TO HIS MAJESTY'S GRENADIER REGIMENT OF FOOT GUARDS.

---

VOL. L.

FROM JULY TO DECEMBER, 1823.

Et quoniam variant morbi, variabimus artes;
Mille mali species, mille salutis erunt.

---

45583

*LONDON:*

PRINTED FOR THE PROPRIETORS,

*By J. and C. Adlard,* 23, *Bartholomew Close*;

PUBLISHED BY J. SOUTER, 73, ST. PAUL'S CHURCH-YARD;

AND MAY BE HAD OF ALL BOOKSELLERS.

WI
LD 31

### INDEX

OF

THE FIFTIETH VOLUME

OF THE

### London Medical and Physical Journal.

PAGE

Absorption, experiments on 6
Aborigines of New Holland, observations on . . . 180
Army Medical Officers' Insurance and Benevolent Fund . 175
Associated Apothecaries, report of 174
Aphonia, case of . . 104
Apparatus, a new one, for the prevention and cure of pointed toe 179
——— for inhaling the vapour of hot water . . . 196
———, galvanic . . 261
Aneurism, inguinal case of . ib.
Artificial palate . . 352
——— pupil, remarks on . 57
Ankle-joint, fracture of . 381
Abdomen, inflammation of . 388
———, encysted tumor in . 443
Arrangement of venous system . 2
Anatomico-pathological researches 25
Arteria innominata tied . 47
Artery, external carotid, ligature of, 48
Ani prolapsus, remarks on . 53
Acupuncturation, remarks on . 57
Article on Pathology . . 518

Books, Catalogue of, 178, 266, 353, 442, 529

Brain, on the structure of . 67

Bronchocele, cases of . . 118

Blue-coloured urine . . 351

Blood urea in the . . 352

Brain, concussion of . . 448

———, encysted tumor in . 467

———, pathology of . . 22

Bile in digestion, effects of . 14

Barcelona fever, remarks on . 33

Bronchotomy, case of . . 49

Biography of Mr. Newby . 177

——— Mr. Mathias . 178

——— Dr. Ryan . 266

——— Dr. Baillie . 353, 436

——— Mr. Houghton . 353

——— Dr. Fawssett . 440

——— Dr. J. N. Johnson 441

PAGE

College of Surgeons (Elections) 174
Catalogue of Books, 178, 266, 353, 442, 529
Correspondents, notices to, 90, 178, 266, 354, 530
Cæsarean section, cases of . 49
Curvature of the Spine . . 91
Cases of Gonorrhœa . . 460
Case of Aphonia . . 104
——— Concussion of the Brain 448
——— Sympathetic Puerperal Convulsion . . . 111
——— Ununited Fracture of Jaw 55
——— Impetigo . . 469
——— Bronchocele . . 118
——— Sarcocele . . 53
——— Hernia Cerebri . 191
——— Extirpation of Uterus . 156
——— Cerebral Inflammation 198
——— Fungus Hæmatodes . 51
——— Tic Douloureux . 201, 271
——— Bronchotomy . . 49
——— Loss of Speech . . 202
——— Inguinal Aneurism . 261
——— Visceral Disease . 267
——— Tetanus . . 446
Combustion, spontaneous . 172
Croup, new treatment of . 258
Calculus, urinary, weighing ten ounces . . . 259
Crania, monstrous . . 350
Croton oil, remarks on . 365
Cavity of the chest, hissing noise in 435
Chest, effects of wounds made in the 12
Canal, digestive, remarks on 17, 26
Condensation of the Gases . 66

Destruction of the Spinal Marrow 80
Derangement, mental, on . 124
Death of a child, supposed from its mother's milk . . 264
Diaphragm, hernia through the 351
Digitalis, a new formula for giving 465
Delirium Ebriositatis, on . 355
Division of the Symphysis Pubes 450

PAGE
Digestion, effects of Bile in . 14
Digestive Canal, remarks on 17, 26
Duct, Thoracic, obliteration of 24
Description of a Rotation Saw . 59
——— the Kystitome Caché ib.

Epidemics and Hygiene, by M. Foderé . . 151, 245
Extraction of the Skeleton of a Fœtus . . . 172
——— a Fork from the Stomach . . . 55
Extirpation of Lower Jaw . 259
——— the Uterus, case of 56
Encysted Tumors, excision of . 260
——— in the Brain . 467
Experiments on the Fusion of Plumbago . . . 261
——— Absorption . 6
Extravasation in Hemiplegia, seat of 351
Ebriositatis, Delirium, on . 355
Exhalation, experiments on . 10
Effects of Wounds made in the Chest 12
——— Putrid Matters . 30
Epilepsy, on Narcotics in the cure of . . . . . 45
Economical method of making Prussic Acid . . . 523
Encyclopædia Londinensis, article on Pathology in . . 518

Fumigating Baths . . 264
Fever and Inflammation, on, by Dr. Lucas . . . 135
———, Barcelona, remarks on . 33
Fusion of Plumbago . . 261
Fractures of the Neck of the Thigh-bone, observations on . 291, 407
Formula, a new one, for giving Digitalis . . . . . 465
Fracture of the Ankle-joint . 381
——— Lower Jaw, ununited, case of . . . . 55
Fungus Hæmatodes, case of . 51
Fork, extraction of, from the Stomach . . . . 55

Galvanic apparatus . . 261
Gases, condensation of . 66
Gonorrhœa, cases of . . 460

Half-yearly Report of Small-pox Hospital . . . 171
Health of the Troops in North Britain, observations on . 275
Hemiplegia, seat of Extravasation in . . . . . 351
Hernia Cerebri, case of . 191
——— through Diaphragm . 351
Hindus, medical and surgical Science of the . . . 451

PAGE
Hissing noise in the cavity of the Chest . . . . 435
Hot water, apparatus for inhaling the vapour of . . . 196
Hydatids, remarks on . . 16

Jaw, ununited fracture, case of 55
Impetigo, case of . . 469
Improved Truss . . 175
Infant, malformation of . 171
Inflammation and Fever, review of Dr. Lucas on . . 135
———, Cerebral, case of 198
——— of the Abdomen 388
Inguinal Aneurism, case of . 261
Insurance Society and Benevolent Fund, report of . . 475
Internal coats of an Artery, rupture of . . . 258
Intussusception, recovery from . 468
Iron, on the employment of, in Utero-gestation . . 435

Kystitome Caché, description of 59

Legal medicine, new questions in 436
Levant and indigenous opium, difference between . . 263
Ligature of external Carotid Artery 48
Lithotomy, remarks on . . 54
Loss of Speech, case of . . 202
Lower Jaw, extirpation of . 259

Malformation of a Male Infant . 171
Materia Medica, Dr. Eberle on 342
Measles, preservative against . 264
Mental Derangement, Dr. Willis on 124
Mercurial Salivation, method of arresting . . . 260
Meteorological Tables 90, 176, 265, 354, 441, 530
Monthly Catalogue of Books, 178, 266, 353, 442, 529

Narcotics in the cure of Epilepsy 45
Neck of the Thigh-bone, on the Fractures of . . 291, 407
Nerves, observations on the . 5
Nervous system, observations on, 3, 422
Neuralgia, cases of . 201, 271
New Holland, on the Aborigines of, 180
Notices to Correspondents, 90, 178, 266, 354, 442, 530

Obliteration of Thoracic Duct . 24
Observations on the Nerves . 5
——— the Tread-mill, 112, 205, 273, 372
——— the Health of the Troops in North Britain . 275
——— in Surgery . 407

PAGE
Observations on Fractures of the Neck of the Thigh-bone . 407
Oil of Croton, remarks on . 365
Operation, Recto-vesical . 55
Operative Surgery, a Treatise on, by Mr. Averill . . 147
Opium, difference between indigenous and Levant . . 263
Ornythohyneus Paradoxus, the spur of . . . . 3

Palate, artificial . . 352
Pathology of the Brain, remarks on 22
———, article on . . 518
Plates, references to . 179, 443
Plumbago, fusion of . . 261
Preservative against Measles . 264
Prevention and cure of Pointed Toe, apparatus for . . 179
Principles of Pathology, review of Dr. Pring on . . 311, 395
Prison Labour, on . . 329
Prolapsus Ani, remarks on . 53
Prussic Acid, economical method of making . . . 523
———, on the use of . 462
Puerperal Convulsion, case of . 111
Pupil, artificial, remarks on . 57
Putrid matters, effects of . 30

Quarterly Reports of Diseases, 169, 347
Question, new, in legal medicine 436

Recto-vesical operation . . 55
Recovery from Intussusception . 468
Renal Inflammation . . 105
Remarks on the Barcelona Fever 33
——— Prolapsus Ani . 53
——— Secale Cornutum . 65
——— Lithotomy . . 54
——— Acupuncturation . 57
——— Artificial Pupil . ib.
Report of the Hospital for Small-pox . . . . . 171
——— Associated Apothecaries . . . . 174
Reports of Diseases . 168, 347
Researches, Anatomico-pathologic 25
Rotation Saw, description of . 59
Rupture of Umbilical Cord . 173
——— the internal Coats of an Artery . . . 258

Salivation, method of arresting . 260
Sarcocele, case of . . 53
Saw, Rotation, description of . 59
Secale Cornutum, remarks on . 65
Skeleton of Fœtus, extraction of 172
Speech, loss of . . . 202
Spinal Marrow, destruction of . 80
Spine, Curvature of . . . 91
Spontaneous Combustion . 172

PAGE
Spur of the Ornythohyneus Paradoxus described . . 3
Stomach, extraction of a Fork from 55
Structure of the Brain, on . 67
Sulphur, a preservative against Measles . . . 264
Surgical Science of the Hindus . 451
Surgeons, College of . . 174
Surgery, observations in . 407
Swelling of Tongue, cured by incisions . . . . 352
Symphysis Pubis, division of the 450
System, the Nervous, observations on . . . 3, 422

Tables, Meteorological, 90, 176, 265, 354, 441, 530
Tetanus, case of . . . 446
Thigh-bone, observations on Fractures of the Neck of the . 291
Thoracic Duct, obliteration of . 24
Tic Douloureux, cases of . 201
Tongue, swelling of, cured by incisions . . . . 352
Tread-mill, observations on, 112, 205, 372
Treatise on Inflammation and Fever, by Dr. Lucas, review of . 135
——— Operative Surgery, by Mr. Averill, review of . 147
Troops, observations on the Health of, in North Britain . . 275
Truss, improved . . . 175
Tuberculous Diseases, remarks on, 15, 23
Tumor, Umbilical . . 257
———, Encysted, in the Abdomen, 443
———, excision of . 260

Umbilical Tumor . . 257
——— Cord, Rupture of . 173
Ununited Fracture of the Jaw, case of . . . 55
Urea in the Blood . . 352
Urinary Calculus, weighing ten ounces . . . 259
Urine, Mr. Howship on the . 226
———, blue-coloured . . 351
Use of Prussic Acid . . 462
Utero-gestation, on the employment of Iron in . . . 435
Uterus and Vagina, double . 1
———, extirpation of . . 56
Vapour of hot Water, apparatus for inhaling . . . 196
Venous System, peculiar arrangement of . . . 2
Veterinary Art, on . . 239
Visceral Disease, case of . 268

Worms, varieties of . . 173
Wounds made in the Chest, effects of, 12

### INDEX.

### REVIEWS.

### DOMESTIC.

PAGE

A Treatise on Mental Derangement, containing the Substance of the Gulstonian Lectures for May, 1822. By F. WILLIS, M.D. Fellow of the Royal College of Physicians . . . . . . . 124

On the Principles of Inflammation and Fever. By C. E. LUCAS, M.D. . 135

A Short Treatise on Operative Surgery, describing the principal Operations as they are practised in England and France; designed for the Use of Students, in Operating on the dead Body. By CHARLES AVERILL, Surgeon, Cheltenham; Fellow of the Royal College of Surgeons . 147

A Treatise on the Epidemic Puerperal Fever, as it appeared in Edinburgh, in 1821-22. To which is added, an Appendix, containing the Essays of the late Dr. GORDON on the Puerperal Fever of Aberdeen, in 1789, 90, 91, 92. By WILLIAM CAMPBELL, M.D. . . . 303, 493

A Treatise on the Disease termed Puerperal Fever; illustrated by numerous Cases and Dissections. By JOHN MACKINTOSH, M.D. . . 219

Report on Puerperal Fever, in Answer to Queries from the Board of Health. By JOHN DOUGLAS, M.D. (Dublin Hospital Reports) . . 303

A Practical Treatise on the Symptoms, Causes, &c. of some of the most important Complaints that affect the Secretion and Excretion of Urine. The whole exhibiting a comprehensive View of the various Diseases of the Kidneys, Bladder, Prostate Gland, and Urethra, illustrated by numerous Cases and Engravings. By JOHN HOWSHIP, Member of the College of Surgeons, London . . . . . . 226

A Series of Elementary Lectures on the Veterinary Art. By Veterinary-Surgeon PERCIVAL, of the Royal Regiment of Artillery . . 239

An Exposition of the Principles of Pathology, and of the Treatment of Diseases. By DANIEL PRING, M.D. . . . . 311, 395

Prison Labour: Correspondence and Communications addressed to His Majesty's Principal Secretary of State for the Home Department, concerning the Introduction of Tread-mills into Prisons; with other Matters connected with the Subject of Prison Discipline. By Sir J. C. HIPPISLEY, Bart. F.R.S. . . . . . . . . . 328

Practical Observations in Surgery. By HENRY EARLE, F.R.S. &c. . 407

Observations on Fractures of the Neck of the Thigh-bone; being an Appendix to the Work on Dislocations and Fractures of the Joints. By Sir ASTLEY COOPER, Bart. F.R.S. &c. . . . . . . ib.

Lectures on the Operative Surgery of the Eye; being the Substance of Part of the Author's Course of Lectures on the Principles and Practice of Surgery which relates to the Diseases of that Organ, &c. By G. J. GUTHRIE, Deputy Inspector of Hospitals, &c. &c. . . . 480

### FOREIGN.

Leçons sur les Epidemics et l'Hygiene publique, faites à la Façulté de Medecine de Strasburg. Par F. E. FODERE . . . 151, 245

A Treatise on the Materia Medica and Therapeutics. By Dr. EBERLE - 342

Mémoire sur quelques Découvertes recentes relative aux Fonctions du System Nerveux; lu a sceance publique de l'Académie des Sciences, le 2 Juin, 1823. Par M. MAGENDIE, &c. &c. . . . . . 422

Considerations, Anatomiques, Physiologiques, et Pathologiques, sur la Luette. Par le Docteur J. LISFRANC, Membre titulaire de l'Academie Royale de Medecine, &c. (Revue Medicale, Juillet) . . . 512

### MISCELLANEOUS.

PAGE

Army Medical Officers' Insurance and Benevolent Fund . . . 175
Article of Pathology in the Encyclopædia Londinensis . . . 518
Case of Mr. MOREL . . . . . . . . 523
Catalogue of Dr. BAILLIE's Papers . . . . . 527
College of Surgeons, Election of officers at the . . . . 174
Dismissal of Mr. HUTCHISON from the Penitentiary . . . 526
Fumigating Baths . . . . . . . . . 264, 525
Half-yearly Report of the Hospital for Casual Small-pox and Vaccination 171
Improved Truss . . . . . . . . 175
Meteorological Society . . . . . . . 434
Report of the Associated Apothecaries . . . . . 174

### OBITUARY.

Death of Mr. NEWBY . . . . . . . 177

——— Mr. MATHIAS . . . . . . . 178

——— Dr. RYAN . . . . . . . 266

——— Dr. BAILLIE . . . . . . 353, 436

——— Mr. HOUGHTON . . . . . . 353

——— Dr. FAWSSETT . . . . . . 440

——— Dr. J. N. JOHNSON . . . . . . 441

### METEOROLOGICAL TABLES.

Pages 90, 176, 265, 354, 441, 530.

### MONTHLY CATALOGUE OF BOOKS.

Pages 178, 266, 353, 442, 529.

### NOTICES TO CORRESPONDENTS.

Pages 90, 178, 266, 354, 442, 530.

### ERRATA.

Pages 354, 530.

### REFERENCES TO PLATES.

Mr. AMESBURY's Instrument for the Pointed Toe . . . . 179

Captain JEKYLL's Vapour-Bath . . . . . . 443

### NAMES OF AUTHORS,

*Of whose Works, Observations, &c. either a detailed Account, or more or less general View, is given in*

### THE FIFTIETH VOLUME OF THE LONDON MEDICAL AND PHYSICAL JOURNAL.

| | PAGE |
|---|---|
| Andral and Breschet, MM. on Hydatids in the Lungs | 16 |
| Ashford, Mr. on Malformation of a Male Infant | 171 |
| Andral, M. on the Digestive Canal | 17, 26 |
| Amesbury's, Mr. Apparatus for Pointed Toe | 179 |
| Amusat, M. his Instrument for breaking Calculi | 54 |
| Averill, M. on Operative Surgery | 147 |
| Bell, Mr. C. on the Nerves | 5 |
| Braun, Dr. on Chlorine in Scarlatina | 522 |
| Bush, Mr. on Morbid Anatomy | 467 |
| Borrone, M. his case of Cæsarean Section | 49 |
| Belcombe, Dr. case of Inflammatory Affection of the Abdomen | 388 |
| Bancal, Dr. on a new Kystitome | 59 |
| Brown, Mr. on Fracture of the Ankle-Joint | 381 |
| Blake, Mr. on Delirium Ebriositatis | 355 |
| Brodie, Mr. on the Effects of Bile | 14 |
| Bampfield, Mr. on Distorted Spine | 172 |
| Beale, Mr. case of Tic Douloureux | 201 |
| Baillie, Dr. Obituary of | 353 |
| Cameron, Dr. his Apparatus for Inhaling Vapour | 196 |
| Cuynat, M. on Elongation of the Uvula | 519 |
| Cloquet, M. case of Blue Urine | 351 |
| ———— case of Hernia through the Diaphragm | ib. |
| Coates, Dr. on Absorption | 6 |
| Campana, M. on the Extraction of Calculi | 55 |
| Cuvier, Baron, on monstrous Crania | 350 |
| Crouzit, M. on Abortion | 523 |
| Colson, M. on Spontaneous Combustion | 172 |
| Craigie, Dr. on the Pathology of the Brain | 22 |
| Cooper, Sir A. Observations on Fractures of the Thigh-bone | 407 |
| Campbell, Dr. on Puerperal Fever | 303, 493 |
| Coindet, Dr. his Formulæ for Iodine | 43 |
| Chaussier, M. on Rupture of the Umbilical Cord | 173 |
| Churchill, Mr. on Acupuncturation | 57 |
| Clark, Mr. on Artificial Palate | 352 |
| Duchateau, Dr. case of Umbilical Tumor | 257 |
| Dupuytren, M. case of Extirpation of the Lower Jaw | 259 |
| ———— on Prolapsus Ani | 53 |
| Duprat, M. on Levant and indigenous Opium | 263 |
| Douglas, Dr. on Puerperal Fever | 303 |
| Dupuy, Mr. on Obliteration of the Œsophagus | 520 |
| Drapés, Dr. his case of Sarcocele | 53 |
| Eberle, Dr. on the Materia Medica | 342 |
| Earle, Mr. observations on Fractures of the Neck of the Thigh-bone | 291 |
| Edwards, Dr. on the Absorption and Exhalation of Azote | 12 |
| Faraday, Mr. on the Condensation of Gases | 66 |
| Faneau de la Cour, M. case of Swelling of the Tongue | 352 |
| Fodoré, M. on Epidemics | 151, 245 |

PAGE

Feville, M. on Extravasation in Hemiplegia . . . . . 351

Flourens, M. on the Nervous System . . . . . . 3

Fodera, M. on Absorption and Exhalation . . . . . 10

Gaillon, M. on the Conferva . . . . . . . 524

Green, Mr. his case of Impetigo . . . . . . . 469

Gaspard, Dr. on the Effects of Putrid Matter . . . . 30

Gunning, Mr. Extraction of the Bones of a Fœtus . . . 172

Goodeve, Mr. on Hernia Cerebri . . . . . . . 191

Good, Dr. on the Tread-Wheel . . . . . . . 205

Gerard, M. case of Urinary Calculus . . . . . . 259

Gregory, Dr. Statistical Reports . . . . . . . 168

——— case of Encysted Tumor in the Abdomen . . . 443

Guthrie, Mr. on the Eye . . . . . . . . 480

Howship, Mr. on the Urine . . . . . . . . 226

Hill, Mr. on the Spur of the Ornythohyncus Paradoxus . . . 3

Hippisley, Sir J. on Prison-Labour . . . . . . 328

——— on the Tread-Mill . . . . . . . 273

Hutchinson, Mr. on the Tread-Mill . . . . . 113, 372

——— on the Employment of Iron during Utero-gestation . 435

Houghton, Mr. Obituary of . . . . . . . . 353

Hufeland, Dr. on Narcotics in the Form of Vapour . . . 45

Harvey, Mr. his case of Tetanus . . . . . . . 446

Hutchison, Mr. his Dismissal from the Penitentiary . . . 526

Jacobson, Dr. on the Venous System . . . . . . 2

Jameson, Mr. his case of Bronchotomy . . . . . . 49

Kinglake, Dr. on Renal Inflammation . . . . . . 105

——— on Croton Oil . . . . . . . . 365

Lavagna, Dr. on Amenorrhœa . . . . . . . 5, 22

Lallemand, M. on Softening of the Brain . . . . . 520

Lisfranc, M. on the Uvula . . . . . . . . 512

——— on Lithotomy . . . . . . . . 54

Lizars, Mr. on Rupture of the Coats of Arteries . . . . 258

Lucas, Dr. on Inflammation and Fever . . . . . . 135

Manchini, Dr. on Division of the Symphisis Pubis . . . 450

Mathias, Mr. Obituary of . . . . . . . . 178

Marshall, Mr. on the Health of the Troops in North Britain . . 275

Mackintosh, Dr. on Puerperal Fever . . . . . . 219

M'Ghie, Mr. on the Excision of Encysted Tumors . . . 260

Magendie, Mr. on the Nervous System . . . . . 422

Marley, Mr. on Cubebs in Gonorrhœa . . . . . 450

Macleod, Dr. on Prussic Acid . . . . . . . 462

Morel, Mr. his Case . . . . . . . . . 525

Mott, Dr. his case of Ligature of the Arteria Innominata . . 47

Newby, Mr. Obituary of . . . . . . . . 177

Nasse, Dr. his case of Obliteration of the Thoracic Duct . . 24

O'Halloran, Dr. on the Barcelona Fever . . . . . 33

Orfila, M. on the Chloruret of Lime counteracting Putrefaction . 528

Painter, Mr. on the Rupture of the Uterus . . . . . 62

Percival, Mr. on the Veterinary Art . . . . . . 239

Physick, Dr. on Fracture of the Jaw . . . . . . 55

Pring, Dr. on Pathology . . . . . . . 311, 395

Partington, Mr. on Aphonia . . . . . . . 104

Paris, Dr. on Medical Jurisprudence . . 119, 208, 295, 390, 474

———, remarks on his Pharmacologia . . . . . 37

Pepys, Mr. Galvanic Apparatus of . . . . . . 261

Pretty, Mr. case of Visceral Disease . . . . . . 267

Prevost, M. on Urea in the Blood . . . . . . 352

——— and Dumas, MM. on Nervous Influence . . . 546

Pessina, M. his method of making Prussic Acid . . . 523

Rolando, Mr. on the Brain . . . . . . . . 67

Rullier, Dr. on the Destruction of the Spinal Marrow . . 80

PAGE

Rolph, Mr. case of Cerebral Inflammation . . . . 198
Recamier, Mr. on Croup . . . . . . . 258
Ryan, Dr. on Loss of Speech . . . . . . 202
———, Dr. Michael, Obituary of . . . . . 266
Rickwood, Mr. on Bronchocele . . . . . . 118
Rosse and Fenoglio, MM. on a hissing Noise in the left Cavity of the Chest, 435
Renaud, M. his case of a Fork extracted from the Stomach . . 55
Stearne, Dr. on the Secale Cornutum . . . . . 65
Sauter, Dr. his case of Excision of the Uterus . . . . 56
Scoutteten, Dr. Anatomico-Pathological Researches . . . 25
Shipman, Mr. his case of Concussion of the Brain . . . 448
Sommé, M. on arresting Salivation . . . . . 260
Sievewright, Mr. on the Medical and Surgical Science of the Hindus . 451
Silliman, Mr. on the Fusion of Plumbago, &c. . . . . 261
Thal, Professor, his Rotation Saw . . . . . 59
Tortual, Dr. on Sulphur as a Preservative against Measles . . 264
————, on the Death of a Child, supposed from its Mother's Milk ib.
Treviranus, M. on the Decussation of the Optic Nerves . . 515
Tiedmann, M. on Double Uterus and Vagina . . . . 1
Travers, Mr. his account of Dr. Pett . . . . . 29
Virey, M. on Intestinal Worms . . . . . . 173
Villerme, M. on Pustules following the Bite of a mad Dog . . 518
Weller, Dr. on Artificial Pupil . . . . . . 57
Williams, Dr. on the Effects of Wounds in the Chest . . . 12
———— on Obstruction of Blood in the Lungs . . . 517
Webster, Dr. his Statistical Report . . . . . 347
Willis, Dr. on Mental Derangement . . . . . 124
Warren, Dr. J. C. case of Inguinal Aneurism . . . . 261
Wishart, Mr. his case of Fungus Hæmatodes of the Eye-ball . . 51

### NOTICES TO CORRESPONDENTS.

*Communications have been received from* Mr. PARTINGTON, Dr. KINGLAKE, Mr. BAMPFIELD, Mr. HUTCHINSON, *and* Mr. RICKWOOD; *and many others are under consideration.*

